# Factors Associated With Symptoms of Depression and Anxiety Among Women Experiencing Homelessness and Unstable Housing During the COVID-19 Pandemic

**DOI:** 10.1001/jamanetworkopen.2021.17035

**Published:** 2021-07-14

**Authors:** Elise D. Riley, Samantha E. Dilworth, Derek D. Satre, Michael J. Silverberg, Torsten B. Neilands, Christina Mangurian, Sheri D. Weiser

**Affiliations:** 1 Department of Medicine, University of California, San Francisco; 2 Department of Psychiatry and Behavioral Sciences, Weill Institute for Neurosciences, University of California, San Francisco; 3 Division of Research, Kaiser Permanente Northern California, Oakland

## Abstract

This cross-sectional study examines symptoms of depression and anxiety among women experiencing homelessness and unstable housing during the COVID-19 pandemic.

## Introduction

The prevalence of depression among individuals in the US has increased 300% during the COVID-19 pandemic, with a greater burden of illness in individuals with lower incomes.^1^ With the goal of informing adaptation of services for socioeconomically marginalized individuals, we surveyed mental health symptoms and social challenges experienced during the COVID-19 pandemic among women experiencing homelessness and unstable housing (HUH). Extrapolating from the available evidence,^2,3^ there are approximately 440 000 women experiencing HUH in the US.

## Methods

Between July and December 2020, we used previously developed methods^3^ to conduct a cross-sectional study among women in San Francisco, California, recruited from homeless shelters, street encampments, free meal programs, and low-income hotels. Eligibility criteria included female sex at birth, age 18 years or older, and a lifetime history of sleeping in public, a shelter, or temporarily with friends or acquaintances (ie, couch-surfing). Interviews, including verbal consent for study participation, were conducted via telephone, and participants were sent $50 reimbursement for completing an interview. Study procedures were approved by the University of California, San Francisco, institutional review board.

Outcome variables included symptoms of depression measured by the Patient Health Questionnaire–9 (range, 0-27, with higher scores indicating more symptoms; moderate-to-severe depression was defined as a score ≥10)^4^ and symptoms of anxiety measured by the Generalized Anxiety Disorder Assessment–7 (range, 0-21 with higher scores indicating more symptoms; moderate-to-severe anxiety was defined as a score ≥10).^5^ We examined associations between depression and anxiety symptoms and challenges experienced since the beginning of the pandemic (March 2020). Covariates included factors previously associated with depression and anxiety: race/ethnicity (self-reported in response to National Institutes of Health categories), recent homelessness (slept in a shelter or in public), unmet subsistence needs (ie, insufficient access to food, clothing, housing, or hygiene resources), social isolation (feeling isolated and unable to rely on others), increased difficulty managing symptoms of a chronic medical condition (HIV, cardiovascular disease, diabetes, asthma, or emphysema), and increased difficulty getting treatment for mental health, substance use, or a chronic medical condition.

Logistic regression was conducted for binary outcomes with Firth penalized likelihood estimation to account for small cell sizes. Linear regression was performed for continuous outcomes. Results from the penalized log likelihood χ^2^ test and the *t* test statistics for logistic and linear regression used 2-tailed tests under an α < .05. Data were analyzed with SAS statistical software version 9.4 (SAS Institute).

## Results

Among 128 study participants (84% of 152 women approached), 51 (40%) were Black, and the median (interquartile range) age was 56 (47-64) years. During the pandemic, 85 participants (66%) had 1 or more unmet subsistence need, 48 (38%) were homeless, and 68 (53%) were socially isolated (Table).

**Table.** Factors Associated With Depression and Anxiety Symptoms During the COVID-19 Pandemic Among Women Experiencing Homelessness and Unstable Housing^a^

Factor	Women, No. (%) (N = 128)	Anxiety^b^		Depression^c^	
		Dichotomous outcome, logistic regression, unadjusted OR (95% CI)	Continuous outcome, linear regression, unadjusted parameter estimate (SD)	Dichotomous outcome, logistic regression, unadjusted OR (95% CI)	Continuous outcome, linear regression, unadjusted parameter estimate (SD)
Age, median (IQR), y	56 (47-64)	0.97 (0.94-0.997)^d^	−0.16 (0.04)^e^	0.96 (0.93-0.99)^d^	−0.14 (0.04)^e^
Race/ethnicity					
White	33 (26)	1 [Reference]	NA	1 [Reference]	NA
Black	51 (40)	1.87 (0.77-4.62)	1.53 (1.28)	1.0 (0.42-2.37)	0.83 (1.36)
Latina	10 (8)	2.70 (0.6-15.92)	3.51 (2.08)	2.40 (0.60-11.30)	1.73 (2.21)
Native American	5 (4)	1.11 (0.19-7.42)	0.01 (2.78)	1.57 (0.27-10.47)	0.83 (2.94)
Asian or Pacific Islander	4 (3)	0.79 (0.11-5.70)	−1.09 (3.06)	0.48 (0.043-3.29)	−1.72 (3.25)
Multiracial	21 (17)	4.20 (1.22-18.25)^d^	3.32 (1.61)^e^	4.36 (1.35-16.48)^d^	2.79 (1.71)
Other^f^	3 (2)	5.56 (0.48-769.85)	8.08 (3.49)^e^	7.84 (0.69-1085.71)	10.03 (3.70)^e^
Recent homelessness (slept in a shelter, on the street, or other public place during the pandemic)	48 (38)	4.61 (1.91-12.72)^d^	5.83 (0.95)^e^	5.20 (2.37-12.19)^d^	4.66 (1.07)^e^
Any unmet subsistence needs	85 (66)	2.18 (1.00-4.74)^d^	3.45 (1.06)^e^	4.81 (2.23-10.81)^d^	5.38 (1.07)^e^
Social isolation	68 (53)	5.22 (2.34-12.38)^d^	5.37 (0.93)^e^	3.29 (1.62-6.88)^d^	5.45 (1.00)^e^
Increased difficulty getting care or medication for a chronic medical condition since the pandemic^g^	28 (22)	3.00 (1.10-10.05)^e^	3.28 (1.23)^e^	6.26 (2.31-20.93)^d^	3.92 (1.29)^e^
Increased difficulty managing symptoms of a chronic medical condition since the pandemic^g^	37 (29)	6.31 (2.18-24.53)^d^	3.49 (1.11)^e^	4.85 (2.06-12.69)^d^	3.66 (1.18)^e^
Increased difficulty getting care or medications for mental health since the pandemic	31 (24)	2.07 (0.83-5.80)	4.56 (1.16)^e^	3.55 (1.48-9.35)^d^	5.27 (1.20)^e^
Increased difficulty getting drug treatment since the pandemic	9 (7)	0.51 (0.14-2.01)	−0.54 (2.05)	0.98 (0.27-3.82)	1.44 (2.16)

Abbreviations: NA, not applicable; OR, odds ratio.

^a^ All measures were based on self-reported survey questions.

^b^ For anxiety, the dichotomous outcome was moderate-to-severe anxiety, defined as a score of 10 or higher on the Generalized Anxiety Disorder Assessment–7. The continuous outcome was score on the Generalized Anxiety Disorder Assessment–7 (range, 0-21, with higher scores indicating more symptoms).

^c^ For depression, the dichotomous outcome was moderate-to-severe depression, defined as a score of 10 or higher on the Patient Health Questionnaire–9. The continuous variable was score on the Patient Health Questionnaire–9 (range, 0-27, with higher scores indicating more symptoms).

^d^ The 95% CI does not include 1.

^e^ *P* < .05.

^f^ Other refers to study participants who gave their race/ethnicity as anything other than White, Black, Latina, Native American, Asian, or Pacific Islander.

^g^ Includes HIV, cardiovascular disease, diabetes, asthma, and emphysema.

Seventy-one participants (55%) had depression and 54 (42%) had anxiety during the pandemic, which is similar to the prepandemic prevalence of symptom scores indicating depression (49% of women) and anxiety (36% of women) in this population.^6^ Factors significantly associated with depression and anxiety included recent homelessness (anxiety odds ratio [OR], 4.61 [95% CI, 1.91-12.72]; depression OR, 5.20 [95% CI, 2.37-12.19]), unmet subsistence needs (anxiety OR, 2.18 [95% CI, 1.00-4.74]; depression OR, 4.81 [95% CI, 2.23-10.81]), and social isolation (anxiety OR, 5.22 [95% CI, 2.34-12.38]; depression OR, 3.29 [95% CI, 1.62-6.88]) (Table). In addition, women with increased difficulties getting care for a chronic medical condition during the pandemic had Patient Health Questionnaire–9 scores that were 3.92 points higher than those who did not have difficulties, and their odds of screening positive for depression were 6-fold higher (OR, 6.26 [95% CI, 2.31-20.93]). Their Generalized Anxiety Disorder Assessment–7 scores were 3.28 points higher, and their odds of screening positive for anxiety were 3-fold higher (OR, 3.00 [95% CI, 1.10-10.05]). Worse mental health outcomes were similarly observed in women with increased difficulties getting mental health care and managing symptoms of a chronic medical condition.

## Discussion

The COVID-19 pandemic has created a parallel mental health crisis that disproportionately affects individuals with low incomes.^1^ Approximately one-half of women experiencing HUH surveyed here experienced depression and/or anxiety symptoms during the pandemic and, in addition to unmet subsistence needs and social isolation, these symptoms were associated with increased challenges accessing non–COVID-19 care and managing symptoms for chronic medical conditions. These findings are from women experiencing HUH, who are among our most socioeconomically disadvantaged citizens and already at high risk for poor health outcomes.^3^ A limitation of the study is that it is a single sample from 1 city, and the effects of COVID-19 may vary by location. However, because San Francisco is a well-resourced city with multiple programs aimed at assisting individuals experiencing HUH, associations between a lack of access to non–COVID-19 health care and mental health conditions are as likely to exist as those reported here and could be more substantial for women experiencing HUH in other US cities. Improving access to basic subsistence needs is critical. It is further critical to decrease barriers to care for chronic medical conditions besides COVID-19, which are strongly tied to mental health yet are in danger of being overlooked and undertreated during the pandemic.
